# Magnetic Resonance Imaging of the Musculoskeletal System at 7T: Morphological Imaging and Beyond

**DOI:** 10.1097/RMR.0000000000000205

**Published:** 2019-04-09

**Authors:** Vladimir Juras, Vladimir Mlynarik, Pavol Szomolanyi, Ladislav Valkovič, Siegfried Trattnig

**Affiliations:** ∗ High-field MR Center, Department of Biomedical Imaging and Image-guided Therapy, Medical University of Vienna, Vienna, Austria; † Oxford Centre for Clinical Magnetic Resonance Research, BHF Centre of Research Excellence, University of Oxford, Oxford, UK; ‡ Department of Imaging Methods, Institute of Measurements Science, Slovak Academy of Sciences, Bratislava, Slovakia; § Austrian Cluster for Tissue Regeneration, Vienna, Austria; || Christian Doppler Laboratory for Clinical Molecular MR Imaging, Vienna, Austria; ¶ Karl Landsteiner Society, St. Pölten, Austria.

**Keywords:** 7 Tesla, cartilage, meniscus, muscle, musculoskeletal, quantitative, tendon, ultrahigh-field

## Abstract

In 2017, a whole-body 7T magnetic resonance imaging (MRI) device was given regulatory approval for clinical use in both the EU and United States for neuro and musculoskeletal applications. As 7 Tesla allows for higher signal-to-noise , which results in higher resolution images than those obtained on lower-field-strength scanners, it has attracted considerable attention from the musculoskeletal field, as evidenced by the increasing number of publications in the last decade. Besides morphological imaging, the quantitative MR methods, such as T_2_, T_2_∗, T_1ρ_ mapping, sodium imaging, chemical-exchange saturation transfer, and spectroscopy, substantially benefit from ultrahigh field scanning. In this review, we provide technical considerations for the individual techniques and an overview of (mostly) clinical applications for the assessment of cartilage, tendon, meniscus, and muscle. The first part of the review is dedicated to morphological applications at 7T, and the second part describes the most recent developments in quantitative MRI at 7T.

Although 7T magnetic resonance (MR) scanners have been in operation in many large hospitals and research centers for more than a decade, only recently have the regulatory authorities given approval for clinical use. The first European regulatory approval, in July 2017, for a clinical 7 Tesla MRI was followed by the U.S. Food and Drug Administration (FDA), which cleared the first clinical 7 Tesla MRI scanner in North America at the Mayo Clinic in October 2017.^1,2^ Musculoskeletal clinical applications are widespread on standard field strengths, such as 1.5 and 3T MR scanners. Ultrahigh field MR scanners provide, however, significant improvements in signal gain, a shortened scan time, or increasing spatial resolution. In theory, as MR signal amplitude is linearly proportional to the net magnetic polarization of the (mostly commonly imaged) hydrogen nuclei, signal should increase by a factor of 2.3 between 3 and 7 Tesla. As the noise also increases slightly between the 2 field strengths (physiological noise increases with the square of the main field strength), the signal-to-noise (SNR) ratio increases approximately by a factor of 2.^3^ This fact can be exploited either by shortening the scan time for particular protocols or by increasing the spatial resolution while maintaining the other parameters, such as field-of-view, the same. This gain is, however, not straightforward, due to inherent obstacles of ultrahigh field MR scanners. Radiofrequency (RF) deposition, measured by specific absorption rate (SAR), is much higher, which results in greater heating of the tissue. B_1_ inhomogeneity is also a considerable problem of ultrahigh field MR scanners, that is, the shortened wavelength (∼15cm) causes difficulties in penetrating the body and the scanner does not work in the near field regime anymore.^3^ In the early days of 7 Tesla, B_1_ inhomogeneity and subsequent challenges in 7T coil construction resulted in the lack of dedicated coils for many tissues and organs of interest. However, recent developments, driven by the demand from the MR community, show a positive outlook. In addition to neuroimaging, spectroscopy, and X- nuclei applications, the musculoskeletal (MSK) system is one of the main targets of ultrahigh-field MR. One of the most frequently imaged MSK tissue is articular cartilage. Increased age of the population and the incidence of obesity have led to one of the most prevalent MSK conditions—osteoarthritis (OA). OA is a complex joint disease that, in addition to cartilage, also affects the menisci, the synovium, and other tissues of the joint. Conventional modalities, such as radiographs and morphological MRI, are reliable for the detection of the symptoms of later OA stages (joint space narrowing or cartilage tissue loss). There is, however, a strong demand for techniques that provide information about the early structural changes of cartilage, thereby offering an opportunity for early interventions. Quantitative MR techniques have a great potential to establish the imaging markers for glycosaminoglycan (GAG) loss and collagen fiber remodeling. Ultrahigh field scanners provide the basis for the improvement of GAG-specific techniques [chemical exchange saturation transfer (gagCEST) and sodium MRI)] and collagen-specific techniques (T_2_ and T_2_∗ mapping, magnetization transfer). Also, focal cartilage lesions after injuries and repair tissue after cartilage repair surgeries are of interest for imaging modalities to avoid the multiple biopsies required for follow-up of patients after treatment. Ultrahigh field MR is very powerful in imaging rapidly relaxing tissues, including tendons, menisci, ligaments, and bones. Higher SNR at 7T enables an accurate estimation of mono- and multicomponent relaxation rates in tendons, calcified cartilage, and bones. Degenerative disk disease (DDD) has received much literature attention in the last several years, as the incidence of intervertebral disk (IVD) disease has steadily increased. As for tendons, in addition to the acute complete tear, other abnormalities, such as tendinopathy, intratendinous necrosis, and partial tears, are relatively common. Skeletal muscle is another important target tissue for MSK imaging and spectroscopy, as many metabolic and degenerative diseases affect the muscle, and thus, dramatically decrease the quality of life for patients. This review summarizes the most recent developments and findings in both morphological and quantitative 7T MR imaging, which improve the diagnosis and treatment monitoring of MSK disorders.

## MORPHOLOGICAL MSK IMAGING AT 7 TESLA

**FIGURE 1:**
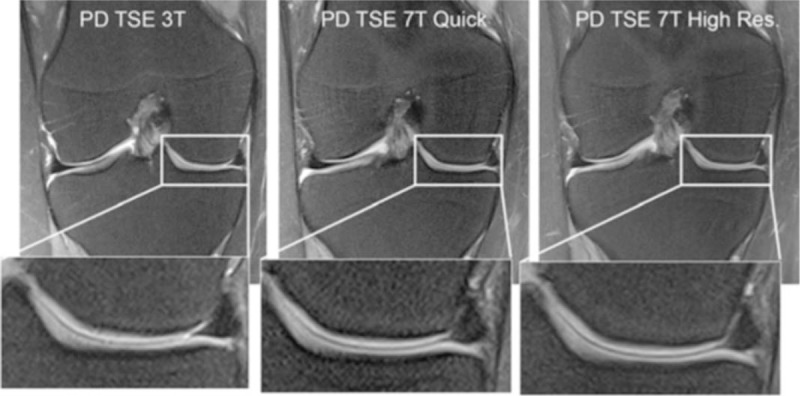
The coronal fat saturated (fs) 2D proton-density turbo-spin-echo (PD TSE) sequence is provided for 3 T (left), 7 T quick (middle), and 7 T high-resolution (right) measurements. The medial femoral condyle with the medial meniscus is enlarged for better visualization of the image quality. Reprinted with permission from *Eur Radiol* 2012; 22:1852–1859.

With the advent of 7T MR scanners, it was important to elucidate the benefits that could be obtained from 7T compared with 3T with respect to scan time reduction or increases in spatial resolution. Welsch et al^4^ compared several protocols and dedicated coils for musculoskeletal MR applications for 3T and 7 T. Two standard 2D sequences (PD-TSE and T_1_-SE) and 3 isotropic 3D sequences (TRUFI, FLASH, and PD-TSE SPACE) were included for comparison. As for acquisition (scan) time (TA), 7T performed generally faster (by a mean factor of 2); TRUFI and FLASH stood out with more than a 3-fold decrease of TA. As for higher spatial resolution, the authors reported better resolution (by a mean factor of 2) with signal-intense sequences, such as the 3D-FLASH sequence, and an almost 4-fold increase in resolution. They concluded that the spatial resolution at 7T can be increased and acquisition time can be reduced, with superior quantitative and comparable qualitative results compared to 3T, but careful optimization must be performed and dedicated coils have to be used (Fig. 1). In addition to the knee joint, Juras et al systematically analyzed the ankle joint at 7T.^5^ They compared 3T and 7T MRI clinical sequences by quantifying SNR and CNR. Ten volunteers were tested using T1-weighted 3D GRE, as well as T_1_-weighted 2D SE and PD-weighted 2D FSE. SNR was calculated for various tissues (cartilage, bone, muscle, synovial fluid, Achilles tendon, and Kager fat-pad). CNR was calculated for cartilage/bone, cartilage/fluid, cartilage/muscle, and muscle/fat-pad. There was a significant increase of SNR at 7T compared with 3T on 3D GRE (60.9%) and 2D TSE sequences (86.7%); however, an SNR decrease was observed with 2D SE (25%). Furthermore, there was a CNR increase on 2D TSE and in the majority of 3D GRE images. These authors underlined the fact that protocol optimization is a must, and the use of dedicated coils is necessary to achieve the best possible results.

With respect to pathologies in joint imaging, Springer et al compared quantitative and semi-quantitative parameters [SNR and diagnostic confidence score (DCS)] between 3T and 7T on 40 patients who had knee pain.^6^ DSC was determined using 5 sequences (2D TSE T2 coronal, 2D TSE PD sagittal, 2D TSE PD FS sagittal + axial, 2D TSE STIR coronal, 2D TSE T1 sag) and SNR was determined by 3 morphological sequences (2D T2 TSE, 2D TSE PD, 3D DESS T2). In a quantitative analysis, all tested sequences provided voxel-volume adjusted SNR higher at 7T than at 3T. DCS was evaluated in 4 anatomically defined areas for potential pathological findings and revealed higher values at 7T than at 3T. In conclusion, 7T improved overall DCS in routine MRI, despite some degradation of images due to chemical shift artifacts in nonfat-saturated sequences. Fine structures in joints, as well as subtle lesions in bone, menisci, and cartilage, were more clearly depicted on 7T.

Benerjee et al investigated GRAPPA-based parallel imaging for high-field MRI of osteoporosis and OA and to ascertain whether implementation of parallel imaging with 7T would require modifications in the calibration or reconstruction strategy.^2^ Considering the limited commercial availability of array coils for MSK applications at 7T, the feasibility of a quadrature coil with 2 elements was evaluated. A modified 3D fs-SPGR, 3D FIESTA-c, and m-bSSFP were used for cartilage. The m-bSSFP sequence was used for the trabecular bone microarchitecture. These authors concluded that the feasibility of in vivo high-resolution MRI at 7T can be considerably increased using parallel imaging because of reduced RF power deposition and increased flexibility in protocol design. Krug et al have investigated the benefits of ultrahigh field MRI at 7T for the examination of knee cartilage and trabecular bone using a fully balanced, steady-state, free-precession (bSSFP) sequence.^3^ They showed that bSSFP and spin-echo imaging is possible at 7T within an SAR limit of 3.2 W/kg. Cartilage SNR of the bSSFP sequence was 2.4 times higher at 7T than at 3T. Trabecular bone SNR increased only minimally compared with 3T. In conclusion, they emphasized that cartilage and bone imaging benefits in SNR increased at 7T. Chang et al have pointed out the problem of trabecular bone microarchitecture with hip imaging at 7T.^4^ The advantages of higher SNR at 7 Tesla have translated into the visualization of individual trabeculae within the proximal femur, employing a T_1_-weighted fast low angle shot 3D FLASH sequence for bone microarchitecture imaging. Furthermore, they devised a T_1_-weighted 3D FLASH with water excitation and volumetric interpolated breath-hold technique (VIBE) with saturation or inversion recovery based, fat suppression for cartilage imaging. Visualization of cartilage, labrum joint capsule, and tendons was achieved using a 2D intermediate-weighted fast spin-echo sequence, which can be readily applied for the clinical imaging of hip. It may allow insight into the pathogenesis of different hip disorders and to detect changes in the hip joint at its earliest stages.

## QUANTITATIVE MRI

### T_2_ Mapping

**FIGURE 2:**
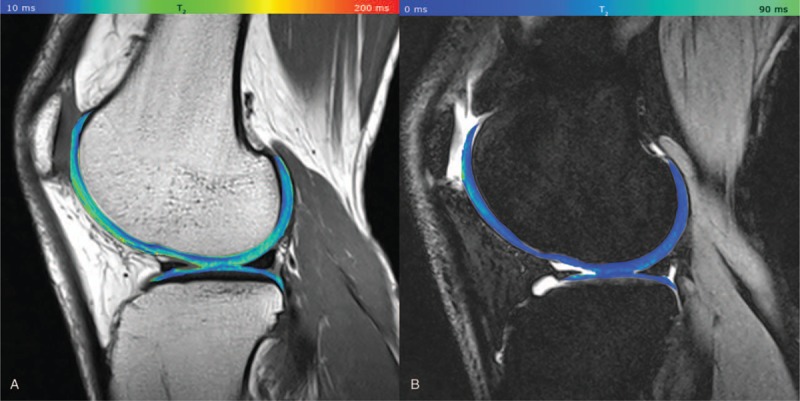
A, T_2_ map acquired by CPMG, pseudo-color-coded, and overlaid on a morphological T_2_-weighted image from a 32-year-old healthy volunteer (TE = 11.9 ms); B, T_2_ map acquired by 3D-TESS, pseudo-color-coded, and overlaid on the second acquired MR image (F_0_). The typical zonal stratification in cartilage is observable with both techniques. However, T_2_ (CPMG) provides apparently higher dynamic contrast of T_2_ values with better depiction of cartilaginous layers. Reprinted with permission from *Eur Radiol* 2016; 26:1905–1912.

Cartilage T_2_ reflects the interaction of water and the extracellular matrix on a molecular level, particularly changes in collagen and water content and anisotropy of tissue.^5^ The orientation dependence of T_2_ relaxation time observed in collagen-rich tissues originates in residual dipolar interactions of water protons due to their restricted motion in the collagen network. Another source of T_2_ anisotropy was attributed to intermolecular dipolar interaction between water protons and those of biopolymers.^6^ In healthy articular cartilage, T_2_ values differ from deep to superficial cartilage layers depending on the collagen orientation. To visualize cartilage zonal stratification in vivo, high in-plane resolution and short scan times are necessary. In general, T_2_ is useful for evaluating cartilage hydration, progression in OA, or assessing damage or cartilage health after physical trauma to a joint, or for surgical procedures.^7–10^ Welsch et al^11^ showed the feasibility of delayed gadolinium-enhanced MRI of cartilage (dGEMRIC), and T_2_ and T_2_∗ mapping to monitor patients after MACT. They observed a significant zonal stratification in healthy cartilage, although almost no stratification in MACT repair sites. In another study from the same group, the authors compared T_2_, T_2_∗, and magnetization ratio between 3 and 7 Tesla.^12^ In 17 knees of healthy volunteers, there were significantly lower global values at 7T than at 3T. The zonal stratification, however, was less pronounced at 7 Tesla and the coefficients of variation, indicating that reproducibility was slightly lower at 7T. As suggested in early works on quantitative MR of cartilage at 7 Tesla, the lower reproducibility was due to the lack of dedicated extremity coils. Chang et al compared a birdcage-transmit, 28-channel receive array with a quadrature volume coil for 7T for T_2_ mapping and they observed 17% to 400% increased SNR/CNR when using a 28-channel knee coil that enabled more reliable morphological imaging and T_2_ mapping.^13^ In thinner cartilage, such as in the hip or ankle joint, where the resolution is even more important, an ultrahigh field scanner may be of great interest. Domayer et al^14^ demonstrated, for the first time, the capability of 7T MRI T_2_ mapping in talus cartilage, even detecting the bilayer zonal pattern of T_2_ in the volunteers in these rather thin cartilage layers compared with the knee joint cartilage layers (Fig. 2). T_2_ values found in the superficial and the deep layer were 39.3  ±  5.9 and 21.1  ±  3.1 ms, respectively. The field-of-view was 140 mm × 140 mm and the matrix was 320 × 320, resulting in an in-plane resolution of 0.4 × 0.4 mm with a slice thickness of 3 mm. Moreover, they compared T_2_ values of 2 different surgical methods: in MFX, T_2_ values in the superficial/deep zone were 43.4  ±  10.5 and 36.3  ±  7.7 ms, and, in MACT, 39.0  ±  9.1 and 27.1 ± 6.6 ms.

The thin cartilage of the hip joint, together with the complex anatomy, is also a challenge in terms of T_2_ mapping. Lazik et al^15^ showed the feasibility of T_2_, T_2_∗ mapping, and dGEMRIC in the hips of 11 healthy volunteers. They used T_2_ and T_2_∗ mapping in the sagittal view with an FOV of 160 × 160 mm^2^, a matrix of 320 × 320, and a slice thickness of 2.5 mm. Echo times used were 10.1, 20.2, 30.3, 40.4, and 50.5 ms for T_2_ mapping (TR 1500 ms) and 3.06, 8.0, 12.94, 17.88, and 22.82 ms for T_2_∗ mapping (TR 130 ms). They found a high correlation between T_2_ and T_2_∗ in acetabular cartilage (*P* = 0.009) and femoral cartilage (*P* = 0.0002) and described regional differences of T_2_ and T_2_∗.^15^

**FIGURE 3:**
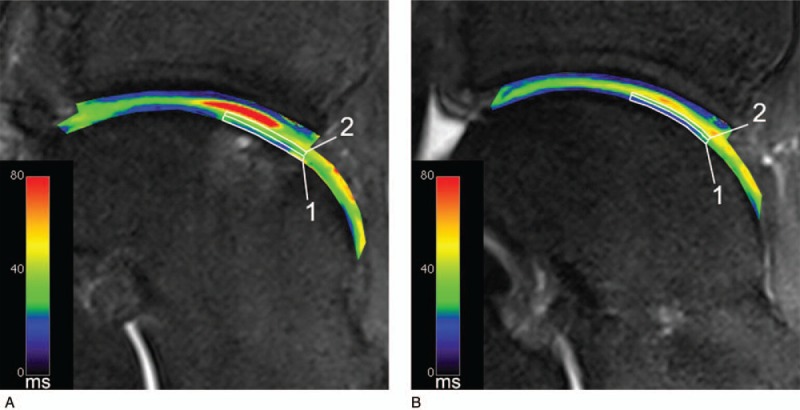
Example of the ROI setting in a case after MACT in the repair site (A) and the reference (B). Both ROIs are set at the same orientation to the static magnetic field. The deep (1) and superficial (2) ROIs are indicated by the white boxes. This case shows T2 values in the repair tissue that are comparable to the articular cartilage both in the deep and the superficial layer. Reprinted with permission from *Osteoarthritis Cartilage* 2012; 20:829–836.

The standard multiecho spin echo (MESE) sequence for T_2_ mapping has many limitations when used at ultrahigh field MR. As it acquires signals from multiple time points along the T_2_ decay for each k-space line during a single repetition time, it suffers from the contamination by stimulated echoes and easily reaches SAR limits due to multiple pulse utilization. A recently introduced triple-echo steady-state sequence (TESS) provides fast T_1_ and T_2_ quantification within a single scan, and, in particular, B_1_-insensitive T_2_ calculation.^16^ Juras et al^17^ used this sequence to estimate T_2_ values in knee cartilage to compare the conventional T_2_ mapping MESE technique with TESS. Using similar sequence parameters for both techniques (slice thickness 3 mm, FOV 160 × 160 mm, matrix size 320 × 320, slice spacing 3 mm), they set up a “standard” TESS protocol with a TA of 4:35 minutes and a “quick” protocol with a TA of 2:05. Although T_2_ values calculated from TESS were generally lower (total mean of 31 ± 5 ms) than MESE (total mean of 46 ± 9 ms), the correlation of T_2_ values between these 2 approaches was very high (ranging from 0.750 to 0.911 depending on the region) (Fig. 3). This study demonstrated the clinical utility of a TESS sequence and concluded that TESS provides results similar to those of a conventional MESE sequence with many benefits, such as shortening of total acquisition time, lowering SAR demands, and insensitivity to B_1_ changes. A similar comparison was done by Kraff and Lazik^18^ in hip joint cartilage with the aim to investigate whether this sequence would be a good candidate for improving workflow in quantitative hip MRI protocols at 7 T. For TESS, these authors used a slice thickness of 2.5 mm, an FOV of 180 × 180 mm, a matrix size of 384 × 384, 32 slices, and a TA of 6 minutes, and, for the MESE, a slice thickness of 2.5 mm, a FOV of 160 × 160 mm, a matrix size of 320 × 320, 6 slices, and a TA of 4:53 minutes. Interestingly, they found much lower correlation coefficients between TESS T_2_ values and those acquired with MESE, ranging from 0.3 to 0.8. T_2_ values were higher when using CPMG—this was attributed to the contributions from stimulated echoes as a result of imperfect refocusing pulses. The authors reported that TESS was most advantageous in clinical studies where the measurement time is a critical factor and where the B_1_ optimization is challenging. Recently, TESS has been used to assess axonal bundles (fascicles) in the median nerve and to determine the normative T_2_ values.^19^ That study found different T_2_ values between 2 subject groups, with significantly higher (*P* = 0.023) values in patients with idiopathic carpal tunnel syndrome (24.27 ± 0.97 ms) than in healthy volunteers (21.01 ±  0.65 ms). It was possible to image the relatively small median nerve due to the high resolution provided by a 7T scanner.

### T_2_∗ Mapping

Some tissues with high organization of collagen fibers, such as tendons, ligaments, and menisci usually appear black on conventional MR images due to the fast transverse relaxation. To acquire signal from these tissues, a very short echo time must be used. In the past, many k-space trajectories that allowed for an ultrashort echo were developed, such as SPRITE,^20^ radial UTE,^21^ 3D cones,^22^ or a stack of spirals (AWSOS).^23^ The 7T scanner enables signal acquisition using ultrashort techniques with higher SNR than clinical scanners. This allows for more robust mono- and bi-exponential T_2_∗ mapping of connective tissues. Juras et al^24^ used 3D-UTE at 7T to demonstrate the regional variation of both mono- and bi-exponential T_2_∗ in the Achilles tendon. They found significant differences between the tendon insertion, the mid portion, and the musculotendinous junction, which were further separated into anterior, posterior, medial, and lateral regions. Moreover, the short T_2_∗ component was found to be a reliable marker for tendinopathies, and thus, may provide additional information to standard clinical imaging in reasonable scan times. Some metabolic diseases, such as diabetes, alter the collagen content and organization in tendons, which can be detected by UTE sequences.^25^ Han et al^26^ compared the performance of the UTE sequence in depicting the tendon microstructure between 3 and 7T. Proper setup of sequence parameters, overcoming increased B_0_ and B_1_ inhomogeneities, and increased RF power deposition using dedicated coils at 7T was necessary to gain higher SNR at 7T versus 3T, as expected. Hager et al^27^ used a micro-MRI insert at 7T to investigate the orientation dependence of T_2_∗ in the knee meniscus. With a resolution approximately 100 times higher than that which is used clinically, they were able to describe the meniscal structures and the strong dependence of T_2_∗ values on orientation toward B_0_. They also reported no bi-exponential decay on a pixel-by-pixel basis. The previously published bi-component T_2_∗ decay in the meniscus probably originated because of the low resolution of analyzed images, which resulted in multiple tissue types contributing to a given voxel.^28^

### T_1_ρ Mapping

Previous works demonstrated the potential of T_1ρ_ to serve as a proteoglycan-specific measure in articular cartilage.^29–31^ Unlike sodium MRI and dGEMRIC, there is no need for special hardware or contrast agent administration while using this technique. The cartilage repair community, however, has been disputing the actual sensitivity of T_1ρ_ to proteoglycans and its clinical applicability for many years. Keenan et al^32^ found, for instance, that T_1ρ_ relaxation time is inversely proportional to proteoglycan content only in cartilage regions with normal T_2_ relaxation. 7T scanners, with higher SNR, and thus, higher resolution, may help investigate this issue further. Singh et al^33^ demonstrated the feasibility of T_1ρ_ mapping at ultrahigh field MR with high SNR (≥ 90), and an in-plane resolution of 0.2 mm^2^, and compared it to 3T measurements. They found 15% lower T_1ρ_ values at 7T than at 3T, with the SNR and resolution substantially in favor of 7T. Moreover, 7T MRI helped to overcome the issues with SAR constraints and B_0_ and B_1_ inhomogeneities. Wyatt et al^34^ studied 20 OA symptomatic subjects to validate 7T T_2_ and T_1ρ_ mapping as markers for differentiating between OA patients and controls. At both field strengths, T_2_ and T_1ρ_ were higher in the lateral femoral condyle and patella in patients with OA, but more regions were significant or approached significance at 7T.^34^

## CHEMICAL EXCHANGE SATURATION TRANSFER

Glycosaminoglycan-specific chemical exchange saturation transfer (gagCEST) is a novel technique, which has the potential to provide information about the amount of GAGs in various cartilaginous structures. It uses selective saturation of the exchangeable hydroxyl protons of GAG,^35^ which exchange with water protons. The performance of this method increases with the magnetic field due to the larger difference in resonance frequencies of the hydroxyl GAG protons and those of water. Along with the larger SNR ratio at 7 Tesla, the larger frequency difference at this field increases the selectivity of saturation and, consequently, the gagCEST effect. The main drawbacks of the gagCEST methodology are the small magnitude of this effect and its sensitivity to subject motion.

The first gagCEST data on articular cartilage at 7T were reported by Schmitt et al^36^ and Sigh et al,^37^ followed by a clinical study that combined 7T gagCEST and sodium MRI in patients with autologous osteochondral transplantation.^38^ Another clinical study of cartilage adjacent to the site of repair surgery showed significant differences in the gagCEST effect compared with reference cartilage.^39^ The gagCEST effect at 7T in the tibio-talar joint was also compared with the T_1ρ_ data.^40^ In a study by Lee et al,^41^ simultaneous saturation with 2 distinct frequencies enabled better isolation of the CEST effect in the knee cartilage. In another methodological study, dynamic correction of the frequency drift was suggested, which enabled the measurement of more realistic gagCEST values.^42^ Parameters for the gagCEST pulse sequence and protocol at 7T were optimized for the best clinical performance in several studies.^43–45^ It was shown that the 7T gagCEST effect in knee cartilage had a moderate-to-good reproducibility when averaged over larger regions of interest. However, a surprisingly large range of the gagCEST values was reported even in healthy cartilage of femoral condyles.^45^ A reason for that might be the variation in the water T2 relaxation time, which can affect the measured gagCEST values in articular cartilage at both 3T and 7T.^46^

## SODIUM MRI

### Technical Considerations

Previously published studies have demonstrated that sodium MR imaging can serve as a noninvasive method for direct determination of the GAG content in cartilage, which plays a crucial role in cartilage homeostasis. New technical developments in the last decade have helped to transfer this method from in vitro to pre-clinical in vivo studies. Sodium imaging has already been applied for the evaluation of cartilage and repair tissue in patients after various cartilage repair surgery techniques and in patients with OA. Sodium MRI, however, is extremely technically challenging. There are a number of reasons for this: first, the sensitivity of sodium MR is only 9.3% of that of proton MR; second, the sodium concentration in healthy cartilage is more than 300 times lower than the concentration of protons; and third, sodium has very short biexponential transverse relaxation times in biological tissues. The resultant SNR is thus more than 3000 times lower in sodium MR than proton one. Ultrahigh field MR scanners equipped with dedicated resonators may help to overcome these challenges; nevertheless, the resolution is much lower and total scan times much longer than those of conventional proton MR imaging. Besides the technical difficulties with sodium MRI, the post-processing of the sodium signal also represents an obstacle to be overcome. The precision of the sodium content measurement is strongly affected by partial volume effects that originate from surrounding tissues, such as bone or synovial fluid. To correctly calculate sodium concentration in articular cartilage, the sodium MR signal must be converted to a sodium concentration. For that, a series of phantoms with known sodium concentration needs to be scanned along with the subject and the calibration curve is calculated by linear fitting of the sodium signal.^47^ As the relaxation times of sodium in healthy and damaged cartilage may vary, this also has to be considered during the evaluation. Chang et al^48^ used a relaxation times (T_1_, T_2_∗_short_, T_2_∗_long_)-corrected method to validate the usefulness of inversion pulses to suppress the synovial fluid in the assessment of native and repair cartilage. In another study, the authors clearly demonstrated a linear increase of SNR between 3T and 7T using a 3D cone pulse sequence.^49^

### Osteoarthritis

**FIGURE 4:**
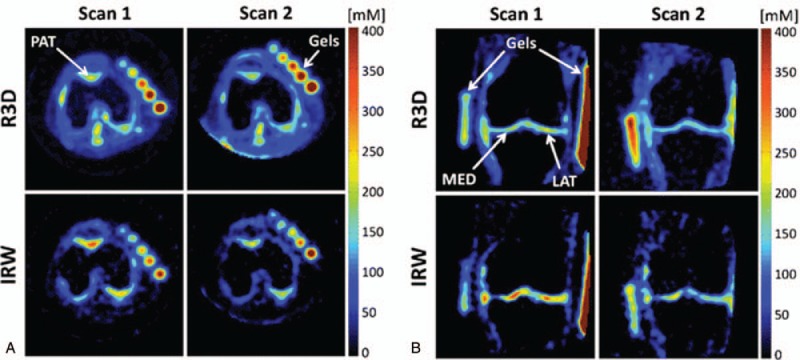
Sodium maps from 1 OA patient, reconstructed from data acquired with fluid suppression (sequence IRW) and without fluid suppression (sequence R3D), at baseline (scan 1) and at the 16-month follow-up (scan 2). A, Transverse slices showing patellar cartilage (PAT). B, Coronal slices showing femorotibial lateral (LAT) and medial (MED) cartilage. The apparent sodium concentrations (ASC) measured with R3D look very similar in both scans, while a higher difference of ASC can be detected visually with IRW in PAT, MED, and LAT. Reprinted with permission from *Radiology* 2018; 28:133–142.

Osteoarthritis is a target disease where sodium MRI can play an important role in diagnosis and treatment monitoring in the future. Early-stage OA assessment, when disease progression might be reversible, is of major interest in cartilage imaging. At the beginning of OA, a repair phase accompanied by hypertrophy occurs, resulting in softening of the articular cartilage due to increased water content as a secondary effect of GAG loss.^50^ To detect this loss with a noninvasive imaging method is extremely desirable. The GAGs contain a high concentration of negatively charged sulfate and carboxyl groups, which results in a negative fixed charge density (FCD) of the articular cartilage. As early as the late 1960s, it was shown that there is a correlation between FCD and sodium concentration as a result of the equilibrium between positively charged sodium ions and negatively charged GAG molecules.^51^ Sodium MRI has great potential to detect subtle GAG content changes due to its specificity to GAG concentration in articular cartilage. One of the first works to discover the link between FCD maps and proteoglycan loss in early-stage OA was published by Wheaton et al^52^ in 2004. Using a 4T MR scanner, they found a mean FCD of −182 mmol/L ± 9 in healthy controls and a decreased FCD with mean values ranging from −108 to −144 mmol/L in symptomatic subjects, which was indicative of proteoglycan loss from the articular cartilage matrix. The first sodium application for articular cartilage on a 7T scanner was reported by Wang et al^53^ in 2009. On 5 controls and on 5 OA patients, they demonstrated the feasibility of sodium MR using a quadrature knee coil and a 3D gradient echo pulse sequence with a radial acquisition. In OA patients, the sodium concentration was reduced significantly by −30% to 60%, depending on the degree of degeneration. Moreover, this was the first study that showed the possibility of reducing sodium MR scan time to a clinically acceptable time of ∼15 minutes. Sodium MRI as an OA marker was validated for the first time in a study involving 19 healthy volunteers and 28 symptomatic patients, using fluid suppression and adiabatic pulses.^54^ Later, the same investigators asked the same patients to undergo a follow-up examination and 12 of the original cohort agreed. The results of this subsequent study demonstrated the sensitivity of sodium MRI to detect the changes in GAG content induced by progressive OA over time.^55^Figure 4 depicts the comparison of the sodium examinations of a patient 16 months apart.

### Cartilage Repair

Cartilage has a very limited capability for self-repair, and the subsequent degeneration often develops into OA. To repair cartilage damage, there are a number of surgical options available, including microfracturing (MFX),^56^ osteochondral graft transplantation,^57^ and autologous chondrocyte implantation (ACI).^58^ The next generation of ACI techniques is often referred to as matrix-associated autologous chondrocyte transplantation (MACT) using hyaluronan, polylactides, or other materials as scaffolds for cell growth.^59^ Maturation of cartilage repair requires multiple follow-ups to monitor the GAG content in the repair site. Sodium MRI was used as a marker for the maturation grading in 2010 by Trattnig et al.^60^ Twelve patients were involved in the study, and all of them were scanned 56 ± 28 months after they underwent a MACT procedure. A 3D GRE pulse sequence with a ^23^Na transmit-receive knee coil was used. A significantly different normalized sodium signal was observed between the cartilage transplant (174 ± 53 a.u.) and healthy cartilage (267 ± 42 a.u.). Subsequently, the same group compared 2 surgical methods for cartilage repair (MFX and MACT) using ^23^Na MRI at 7 Tesla.^61^ The study involved 9 MFX patients and 9 MACT patients, with postoperative intervals of 33.5 ± 25.3 and 33.2 ± 25.7 months for MFX and MACT, respectively. The results showed that the normalized sodium signal was significantly lower in BMS and MACT repair tissue, compared with reference cartilage. Moreover, the normalized sodium was significantly higher in MACT than in MFX repair tissue, suggesting better quality in MACT than in MFX. In another study by Krusche-Mandl et al,^38^ the authors followed-up patients 8 years after autologous osteochondral transplantation using gagCEST, sodium MRI, and T_2_ mapping. Interestingly, of the 3 biochemical MR methods, only T_2_ correlated with clinical outcome. Technical developments in sodium MR allowed acquisition of the signal from joints with thinner cartilage, as well, such as the ankle joint. Zbyn et al^62^ demonstrated the feasibility of ^23^Na imaging for monitoring patients after MACT and MFX in the ankle and subtalar joints.

### Sodium MRI of Tendons and Spine

**FIGURE 5:**
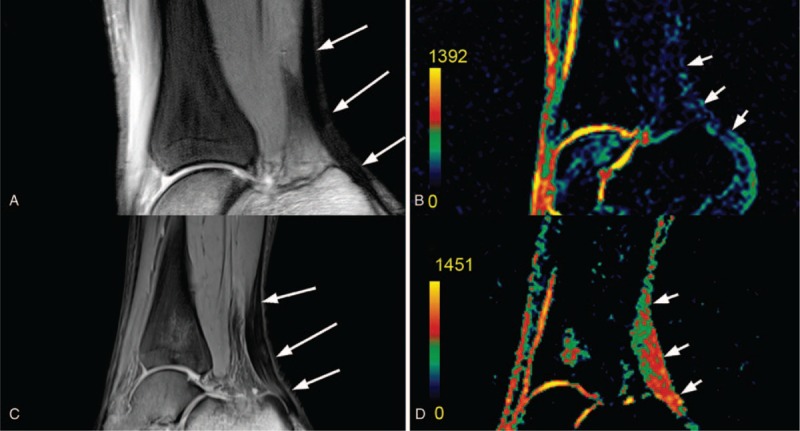
MR images in a 27-year-old healthy volunteer. A, Proton-density-weighted, 2-dimensional, turbo spin-echo image depicts the centers of each ROI (arrows). B, Corresponding color-coded, 3-dimensional, gradient-echo sodium image depicts Achilles tendon sodium signal (arrows), which is minimal. MR images in a 46-year-old patient with chronic Achilles tendinopathy. C, Proton-density-weighted, 2-dimensional, turbo spin-echo image depicts centers of each ROI (arrows). D, Corresponding color-coded, 3-dimensional, gradient-echo sodium image depicts Achilles tendon sodium signal (arrows), which is obviously higher than that in the healthy volunteer. The sodium signal increase was observed in the whole tendon, and not only in regions with clinical findings (middle region in this case). All patients had focal thickening of the Achilles tendon without a fluid-like signal intensity increase on proton-density-weighted, 2-dimensional, turbo spin-echo images. The color scale represents sodium signal intensity values. Reprinted with permission from *Radiology* 2012; 262:199–205.

Sodium MRI has also found a broader application for other structures, such as tendons and IVDs. The normalized sodium MR signal may serve as a marker for patients with Achilles tendinopathy by detecting the increased proteosynthesis (which results in increased GAG content) of the affected tendon. Juras et al used 7T whole-body MRI with a 15-channel sodium knee coil to investigate 8 patients with tendinopathy and 20 controls. They found increased sodium SNR in patients (9.3 ± 2.3), indicating an elevated GAG content, compared with healthy controls (4.9 ± 2.1) (Fig. 5).^63^ The same group investigated sodium MRI as a potential marker for biochemical changes induced by side effects in the Achilles tendon after ciprofloxacin intake.^64^ It has been reported in the orthopedic community that the intake of ciprofloxacin may lead to tendon harm with the development of tendinopathies and even partial or complete tendon tears. They scanned patients 10 days before the ciprofloxacin treatment, and 10 days and 5 months after treatment. The mean sodium signal decreased significantly, by almost 25%, in the whole tendon [from baseline to 10 days after ciprofloxacin intake, 130 arbitrary units (au) ± 8 to 98 au ± 5, respectively; *P* = 0.023] and returned closer to baseline after 5 months (116  au ± 10). A similar behavior of the sodium signal was also found in tendon insertion. This study demonstrated a ciprofloxacin-induced reversible decrease of the normalized sodium MR imaging signal in the Achilles tendon of healthy volunteers.

The IVD containing GAG is also an interesting tissue for sodium MRI. Noebauer-Huhmann et al^65^ performed a study in 10 asymptomatic volunteers (mean age, 30 years; range, 23 to 43 years) to validate the feasibility of sodium MR in IVD and to find the correlation between normalized sodium signal intensity and T_2_ mapping/Pfirrmann score. The Pearson coefficient showed a correlation between sodium imaging and the Pfirrmann score, a moderate negative correlation between T_2_ values and the Pfirrmann score (*r* = -0.62), and no correlation between sodium imaging and T_2_ mapping (*r* = 0.06). This was the first study to demonstrate the feasibility of in vivo 7T MR sodium imaging of the IVD, and to determine the link between quantitative values and clinical score. This might be interesting for future in vivo studies of disk composition and the mechanisms of degenerative disk disease.

### Future Outlook

Modern methods, such as machine-learning, may play a crucial role in sodium MRI evaluation and bring it closer to clinical use by overcoming the tedious manual assessment of low-resolution and low SNR sodium images. Madelin et al^66^ at New York University recently suggested a machine-learning method for the assessment of the possible utility of machine-learning to classify subjects with and subjects without OA. In this study of 19 controls and 28 OA patients, they were able to isolate the best OA predictors with an accuracy as high as 74%.^66^ In other work by the same group, compressed sensing was applied to under-sample a 3D radial pulse sequence for sodium MRI at 7 Tesla. With this technique, it is possible to decrease the scan time by a factor of 2 without losing accuracy in sodium concentration detection over different regions in order to detect early signs of OA. The best results were achieved by a combination of compressed sensing and nonuniform Fast Fourier Transform (NUFFT).

### MRI and MRS of Muscle

Muscle MRI provides valuable information about many aspects of the structure and function of skeletal muscles, such as the presence of edema or fatty infiltration, or changes in mitochondrial metabolism or tissue perfusion. This makes muscle MRI a very powerful tool in the diagnosis and follow-up of patients with several muscle diseases, for example, muscular dystrophies,^67^ and/or whole-body metabolic disorders.^68^ The next few paragraphs will describe several muscle MRI techniques that benefit from the increased SNR of ultrahigh field systems.

### Structural Imaging

Muscle structural MRI is primarily focused not only on detecting edema and fatty infiltration^67,69^ but can be also used to identify skeletal muscle denervation^70^ or measure the muscle architecture.^71^ The fatty infiltration observed in muscle MRI is of particular interest in limb-girdle muscle dystrophies, as this has been shown to correlate with muscle strength and functional status.^72^

Fatty infiltration can be identified by T_1_-weighted imaging, the quantification of which can be challenging,^73^ or, by the more quantitative Dixon technique.^74^ The Dixon technique utilizes the in-phase/out-of-phase cycling of fat and water. The in-phase and out-of-phase images can be combined to create fat-only and water-only images and provide a proton density fat fraction.^75^ Alternatively, proton magnetic resonance spectroscopic imaging (^1^H-MRSI) can be used to define not only fatty infiltration of muscle tissue but also of muscle cells themselves,^76^ which is particularly important in type 2 diabetes mellitus.^77^ The separation of extra- and intramyocellular lipids grows larger at ultrahigh fields and the increase in SNR permits high spatial resolution acquisition in a reasonable time.^78^

Edema and denervation are quite identifiable on T_2_-weighted images, and better still on short inversion time inversion-recovery (STIR) MR images.^67,70^ As the chemical shift separation between fat and water is pronounced at ultrahigh fields, the length of fat saturation pulses can be substantially shortened.^79^ Note that simple qualitative or semi-quantitative comparisons could lead to data misinterpretation, and thus, proper quantitation methods have to be applied to provide information about disease severity.^80^

Direct information about the spatial architecture of skeletal muscle tissue can be obtained by diffusion-weighted MRI, which exploits the restricted diffusion of water molecules along the muscle fibers. Acquiring several diffusion-weighted images allows full characterization of the diffusion tensor.^81^ The diffusion tensor imaging (DTI) approach yields a number of indices that closely define the anisotropic organization of skeletal muscle tissue,^71^ providing an interesting tool for the investigation of muscle injuries.^82^ It has been demonstrated that the reliability of estimating DTI parameters is substantially affected by the SNR and the percentage of fat composition (%fat).^83,84^ In particular, low SNR data may result in an overestimation of the eigenvalues and an underestimation of the mean diffusivity, making DTI a very good target for ultrahigh field MRI, which provides increased SNR.^85,86^

### Functional Imaging

In addition to structural information, muscle MRI also provides information about muscle energy metabolism through the utilization of phosphorus (^31^P) MR.^87^ The high-energy phosphate bonds in adenosine-triphosphate (ATP) and phosphocreatine, both readily detectable by ^31^P-MRS, serve as an energy source for muscle cells and for the rapidly available energy reserve for ATP resynthesis, respectively. Furthermore, by exploiting the chemical shift difference between PCr and inorganic phosphate (Pi) resonances, intramyocellular pH can be calculated.^88^ But, the true strength of ^31^P-MR of skeletal muscles lies in the fact that it allows noninvasive assessment of the rates of chemical reactions involved in energy metabolism.^89^ Of particular interest is the possibility to investigate the oxidative energy production by muscle mitochondria during exercise and subsequent recovery.^87,89^ This has been repeatedly shown to be impaired in patients with muscle dystrophies,^90^ peripheral arterial disease,^91,92^ heart failure,^93^ and in systemic disease, such as obesity^94^ and type 1 and 2 diabetes mellitus,^95,96^ and is also of great interest in sports medicine.^68^

**FIGURE 6:**
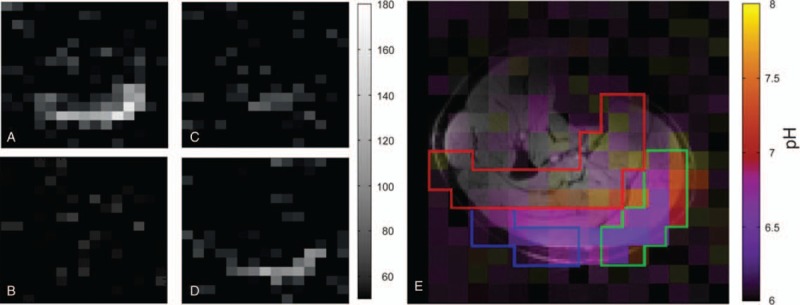
^31^P real-value MR images acquired in a subject during the exercise-recovery experiment (averaged over 3 acquisitions). PCr (A) and Pi (B) at rest. The lower PCr signal during exercise (C) is accompanied by a detectable Pi signal (D) in the gastrocnemius muscles. E, A pH image obtained during exercise (averaged over 5 acquisitions) showing different pH values within the depicted ROIs of the examined muscle groups (gastrocnemius medialis, green; gastrocnemius lateralis, blue; and soleus, red), overlaid over a water image. Reprinted with permission from *Magn Reson Med* 2016; 75:2324–2331.

The inherently low SNR of ^31^P-MR on clinical scanners typically leads to the use of surface coils and limits the choice of sequences to nonlocalized acquisitions.^87^ However, it has been recently shown that such rough localization is not sufficient for exercise-recovery studies, if several differently active muscle groups are detected simultaneously.^97,98^ The use of ultrahigh fields significantly improves the SNR of ^31^P-MR, allowing advanced multi-voxel localization schemes with sufficient temporal resolution to be used for dynamic studies.^99–102^ High spatial resolution can be achieved through spectrally selective MRI; however, it is typically limited to PCr dynamics measurement only,^99^ and losing all other information present in the spectra. Although recent developments have allowed the information about intramyocellular pH to be extracted from ^31^P-MRI as well,^100,103^ still, more information can be acquired from the acquisition of full ^31^P spectra (Fig. 6). This can be achieved either by acquiring a rapidly sampled MRSI matrix^101^ or by interleaving 2 single-voxel excitations.^102^

The ability of skeletal muscle mitochondria to synthesize ATP is highly reliant on oxygen supply. Therefore, the dynamics of muscle tissue perfusion, quantifiable by arterial spin labeling (ASL), are of great interest, particularly in patients with peripheral arterial disease.^104^ Signal decay during inflow time due to T_1_ relaxation, and low SNR, often limit ASL investigations. The use of ultrahigh fields mitigates both these limitations^105^ and allows the combination with T_2_∗-weighted imaging that provides complementary information about blood oxygenation.^106^

Muscular dystrophies, inherited myopathies, muscle injury, as well as metabolic diseases can be identified and their treatment monitored by skeletal muscle MRI. Both structural and functional changes can be quantified, and together, provide complex information about muscle health. Increased SNR and chemical shift separation of ultrahigh fields significantly increase the accuracy, specificity, and reproducibility of muscle MRI examinations.

## CONCLUSION

The higher SNR, more reliable quantitative techniques, the improved contrast, and better X-nuclei MR capability at 7T translate into better spatiotemporal resolution and enhanced biochemical and metabolic tissue characterization. Thus, 7 Tesla MR imaging improves anatomic detail and supports a higher degree of diagnostic confidence when imaging cartilage and other joint tissues. Recent developments in quantitative MR techniques at 7T show the potential to include such techniques into routine clinical imaging, thus helping to merge morphological and compositional information. Clinically approved 7 Tesla MRI scanners will definitely propel the innovations in medical imaging in the future.
